# Development of the Short-Form Yin Deficiency Scale Using Three Item Reduction Approaches

**DOI:** 10.1155/2024/5533815

**Published:** 2024-01-19

**Authors:** Young-Jae Park, Ji-Myung Ok

**Affiliations:** ^1^Department of Biofunctional Medicine and Diagnostics, College of Korean Medicine, Kyung Hee University, Seoul, Republic of Korea; ^2^Department of Diagnosis and Biofunctional Medicine, Kyung Hee University Hospital at Gangdong, Seoul, Republic of Korea; ^3^Department of Human Informatics of Korean Medicine, Graduate School, College of Korean Medicine, Kyung Hee University, Seoul, Republic of Korea

## Abstract

**Background:**

Yin deficiency (YD) is a pathological condition characterized by emaciation, afternoon fever, dry mouth, and night sweats. The incidence of YD is 23.3%. A 27-item Yin Deficiency Scale (YDS) was developed to estimate the clinical severity of YD. This study aimed to develop three short-form YDS versions to reduce the burden of response time, using three item-reduction approaches: Rasch, equidiscriminatory item-total correlation (EITC), and factor-based analyses.

**Methods:**

Two datasets were analyzed from previous studies (169 outpatients from May to June 2009 and 237 healthy college students from January to April 2016). The optimal response category was examined using Rasch analysis. Items with higher item-total correlations were determined using the EITC. Using a factor-based approach, the items were reduced, while maintaining the original YDS construct. Reliability was estimated using the person separation index (PSI) and Cronbach's *α* values. The predictive accuracy was examined using the area under the curve (AUC). Finally, the relationship between YD and dysfunctional breathing (DB) was examined using factor scores from the YDS and the Korean version of the Nijmegen Questionnaire (KNQ).

**Results:**

We developed two 14-item YDS versions using the Rasch and EITC approaches, and a 16-item YDS version using a factor-based approach. Rasch analysis suggested an optimal response category of five points. The PSI of Rasch and Cronbach's *α* of the EITC and factor-based versions were 2.19, 0.855, and 0.827. The AUCs of the three short-form YDS were 0.812, 0.811, and 0.818. The sensitivity of the EITC-YDS was 0.632, which was lower than its specificity of 0.875. The fatigue-related scores of the factor-based YDS were fairly correlated with the factor scores of the KNQ estimating the DB (*r* = 0.349–0.499).

**Conclusion:**

The 14-item Rasch- and 16-item factor-based YDS may replace the original YDS during YD's primary screening, epidemiological surveys, and health checkups.

## 1. Introduction

Yin deficiency (YD) is a pathological condition with diverse symptoms and signs, including emaciation, fatigue, pain, and weakness, especially in the lower limbs; afternoon or night coughs; dry mouth; night sweating; and frequent urination [1]. A previous study reported that the incidence of YD was 23.3% in the elderly group [2]. YD is induced by insufficient yin fluid, including intra- and extracellular fluid, lymphatics, blood, and synovial fluid, and thus, the diminished moisturizing function may secondarily result in heat- or dryness-related symptoms and signs [3]. Some studies have reported that YD is a subtype of the pathological patterns of climacteric syndrome [4], tuberculosis [5], diabetes mellitus [6], and psychiatric disorders [7]. Increased YD was associated with the survival rate in late-stage cancer [8]. Considering the incidence of YD and its broad physiological and pathological spectrum, a questionnaire that can initially screen for the presence or absence of YD will be helpful for clinical trials, epidemiological surveys, and health checkups.

Park et al. developed the 27-item Yin Deficiency Scale (YDS) [9]. The YDS consists of eight factors with a Cronbach's *α* of 0.885 [9]. Based on receiver operating characteristic (ROC) curve analysis, the predictive accuracy of the YDS estimated by the area under the curve (AUC) was 0.875, and its cutoff value was determined as ten points. Since its development, the 27-item YDS has been widely used to evaluate the clinical severity of YD. YD scores estimated by the YDS were associated with an aggravation of the quality of life [9]. Increased YDS scores were associated with decreased blueness of the face and tongue tip [10, 11]. Regarding vocal quality, increased YDS scores were associated with decreased modulation of the fundamental frequency [12]. The YDS scores for the young population with dysfunctional breathing (DB) were higher than those without DB [13]. Although the YDS has been broadly utilized to estimate the clinical severity of YD, it has 27 items, which may require response time and the ability to complete it [14]. In particular, patients with difficulty with handwriting or cognition may be affected by the length of the different questionnaires [14]. Therefore, this study aimed to reduce the number of items in the original YDS using three item reduction methods: Rasch, equidiscriminatory item-total correlation (EITC), and factor analyses.

Rasch's analysis is based on item response theory, in which each item response in the questionnaire is taken as an outcome of the independent interaction between the respondents' abilities and item difficulty [15]. To overcome the limitations of classical test theory, Rasch analysis includes an examination of item hierarchy, fitting error, and differential item functioning (DIF) by sex and age [14, 16]. EITC is a modified version of item-total correlation (ITC) [17]. In the EITC, items are discriminated through three percentile points (25%, 50%, and 75%) of the total scores, and correlations between the dichotomous scores of the items and the total scores of the YDS may be calculated within the three percentile categories [18, 19]. The third approach is factor-based item reduction. The Korean version of the Nijmegen Questionnaire (KNQ), which assesses DB-related symptoms, comprises four etiological factors [20]. If the number of YDS items is reduced while maintaining the construct of factors and reliability levels, it will be helpful to understand the relationship between the etiology of YD and DB by examining the correlations between the short-form YDS and the factors of the KNQ.

In summary, Rasch analysis minimizes bias due to item hierarchy and DIF by sex and age, whereas EITC and factor analyses reduce item numbers while maintaining the reliability and construct validity of the original questionnaire. By comparing the advantages of these three item-reduction approaches, researchers and clinicians may be able to relieve the burden of time or handwriting of respondents through a short-form questionnaire that suits their purposes. Finally, we calculated the AUC of the three short-form YDS versions using receiver operating characteristic (ROC) curve analysis and compared their predictive accuracies with the 27-item YDS.

## 2. Methods

### 2.1. Data Sources

Two datasets were used in the study. One dataset was previously used to develop the original version of YDS [1]. In the previous study, 169 outpatients (39 men aged 42.1 ± 14.7 years; 130 women aged 43.5 ± 14.9 years) from 12 Korean medical clinics completed the 27-item YDS from May to June 2009 [1]. Twelve Korean medical doctors with clinical experience, blinded to the YDS scores, determined the presence or absence of the YD pattern for each outpatient. Another dataset was collected from 237 college students (130 men aged 21.4 ± 1.9 years; 107 women aged 21.4 ± 3.0 years) who had no impediments to daily life caused by psychological or respiratory problems from January to April 2016, and they were asked to complete the KNQ, together with the 27-item YDS [13]. In the two datasets, the YDS items were rated on a 7-point Likert scale: 1 = disagree very strongly; 2 = disagree strongly; 3 = disagree; 4 = neither agree nor disagree; 5 = agree; 6 = agree strongly; 7 = agree very strongly. The items of the KNQ are rated on a 5-point Likert scale: 0 = never; 1 = rarely; 2 = sometimes; 3 = often; 4 = very often. The second dataset did not include information on the clinicians' determination of YD and was used only to examine the relationship between the factor scores of the short-form YDS and the KNQ. The study protocol was approved by the Institutional Review Board of Kyung Hee University Oriental Medical Hospital at Gangdong (approval number: KHNMCOH 2021-02-001).

### 2.2. Rasch Analysis

Rasch analysis used the partial credit model because the 27-item YDS was answered using polytomous responses such as a 7-point Likert scale [15]. The first step in Rasch analysis was to evaluate the appropriateness of the response category. Category probability curves and the ordering of the response categories were examined. If the peak of one curve overlapped with another peak, the response category was excessive, and one of the two response categories was removed [15]. Along with examining category probability curves, the ordering of response categories was examined using step calibration values. Despite the well-separated peaks of each probability curve, the disordered step calibration value, the decreased calibration value among all other increased calibration values, indicated an excessive response category and the category needed to be fused with the adjacent category. Therefore, the examination of the optimal response category was repeated until the separation of probability curves and the ordering of calibration values was satisfied. The second step in Rasch analysis was to examine the DIF. DIF analysis is a measurement of bias and refers to the difference in the probability of providing a certain response between groups [16]. In most cases, age and sex differences result in DIF [16, 21]. Therefore, differences in the item responses of the YDS between the sexes and between the older and younger age groups were examined. Rasch modeling assumes that items are weighted according to their difficulty along a linear logistic function, and the mean square (MnSq) levels and chi-square statistics divided by the degrees of freedom are calculated to examine whether the difficulty of each item fits the linear function [14]. Therefore, the third step was to evaluate the MnSq levels of infit and outfit for each item [18]. Through the evaluation of MnSq levels, misfitting items were deleted, and iterations of fit evaluations were conducted until all items were free of fitting errors [18]. Finally, the unidimensionality and reliability of Rasch YDS were examined [22, 23].

### 2.3. EITC

EITC, a modified ITC, was used to reduce items in the questionnaire [24]. ITC focuses on the correlations between each item's scores and the questionnaire's total scores. The EITC reset the three cutoff points according to the three percentile levels of the total scores (25%, 50%, and 75%) and transformed the total scores into dichotomous values [18, 19]. For example, total scores below and above the cutoff point of 25% were transformed into scores of 0 and 1. Similarly, other dichotomous total scores were determined according to 50% and 75% cutoff points. Thereafter, the EITC was calculated as the correlation between the three sets of dichotomous value-transformed total scores and the questionnaire item scores. Three sets of tables according to the three percentile categories were rearranged in descending order according to the EITC score. Four or five items with the top-ranked EITC were extracted from the 25% percentile category. The same number of items with the top-ranked IETC as those in the 25% percentile category were extracted from the 50% and 75% percentile categories. If the same item was on the top list for both the 25% and 50% categories, it was dropped from the list of the 50% group, and the next-ranked item from that group was substituted into the 50% list. The item in the 75% list was dropped, and the next-ranked item was substituted if it was in both 50% and 75% ranks [18, 19]. As it was reported that Cronbach's *α* > 0.800 is preferable [25], we calculated the minimal item numbers to guarantee a Cronbach's *α* of 0.800 using the Spearman–Brown prophecy formula [26]. If the total number of items to satisfy Cronbach's *α* level is a multiple of three, all top-ranked items may be extracted from the three percentile groups. However, if the total number is not a multiple of three, the multiple of three items exceeding the minimal numbers suggested by the Spearman–Brown prophecy was primarily extracted from the three percentile groups, and the items with the lowest EITC were removed until the minimum item numbers satisfying Cronbach's *α* of 0.800 were reached.

### 2.4. Factor Analysis

The items of the original YDS were previously determined using the contribution scores to YD by 50 Korean medical clinicians who were asked to rate forty-three items on a 7-point Likert scale (1 = no contribution to YD; 7 = greatest contribution to YD) using the Delphi method [27]. Through two iterations of feedback, 30 items with a contribution score over 4.00 were extracted, and the following study finally determined the 27-item YDS satisfying reliability and construct validity [1].  Table 1 lists the final 27 items and mean contribution scores for YD estimated by clinicians [27], and Supplementary Table S1 lists eight factors of the YDS extracted from the 27 items using principal component analysis (PCA) [1]. As shown in Supplementary Table S1, eight factors were associated with the symptoms, lesions, and subtypes of YD. For example, cough, fever, pain, and fatigue were named according to the symptoms of YD, whereas urine and skin factors were named according to the lesions affected by YD. Kidney liver deficiency is one of the most frequently observed subtypes of YD in clinical cases.

As mentioned earlier, we speculated that a factor-based approach may help examine the relationship between the symptoms, lesions, and subtypes of YD and the clinical severity of the disease. A factor-based approach was implemented using the four-step item-reduction procedure proposed by Smith et al. [28]. This procedure had the advantage of minimizing the loss of reliability level while maintaining the construct of factors. In Step 1, Cronbach's *α* values of all factors and whether the values may increase when an item is removed from each factor were examined. In Step 2, we examined whether the decrease in Cronbach's *α* values may be minimized when removing an item. In Step 3, we examined whether face or content validity was maintained after the items were removed or retained in Steps 1 and 2. If face or content validity collapses, returning to Steps 1 and 2, some items may be retained despite their low reliability. In Step 4, a principal component analysis was conducted to examine whether there were remarkable changes in the construct for the reduced items. However, we omitted step 3 from the development of the short-form YDS because all items of the original YDS satisfied content validity via experts' contribution scores for the YD. In other words, it was inappropriate to add dropped items to maintain or increase the validity level. Therefore, we conducted factor-based item reduction, where Cronbach's *α* values of the eight factors of the original YDS were examined, and the items contributing to an increase in overall Cronbach's *α* value (step 1) or contributing to a minimal decrease in the value (step 2), were removed until two items remained with each factor. Thereafter, a PCA was conducted on the short-form YDS to examine whether there were any changes to the construct of the original YDS (Step 4). Finally, the overall Cronbach's *α* coefficient of the short-form YDS was compared to that of the 27-item YDS.

### 2.5. Statistical Analysis

#### 2.5.1. Rasch Analysis

In examining the response category, the ordering of the item responses was acceptable when the total counting numbers of each response category were higher than 10 points, the average measure and step calibration showed an ascending order, and the outfit level of each category was lower than 2.0 [29]. If there was any violation among the item response categories, the category was unified with an adjacent category, and examination of the ordering of all categories was repeated until the violations were corrected. Together with the numerical examination, the overlap of a category curve peak with other curves was examined, and the fusion of two adjacent categories was repeated until all the peaks of the category curves were well separated [30]. DIF was assessed in both sex and age groups. The median value of the participants' age was 41.0 years, thus those >41 years to the older group and those <41 years to the younger group. Differences in logits between men and women and between the older and younger groups were examined using the chi-square test [16]. Infit and outfit were assessed, and items with MnSq values of infit or outfit <0.70 or ≥1.40 were removed sequentially [18]. From the item response perspective, unidimensionality denotes that, among the short-form YDS, the second factor may comprise only one item, which helps avoid scoring unrelated dimensions within the reduced items [22]. Unidimensionality was determined when the unexplained variance in the first contrast extracted from the PCA was <2.0 [22]. Person separation and reliability indexes were calculated to examine the reliability level of the reduced items where separation index ≥2.0, or reliability index ≥0.8, was considered high-reliability levels [31].

#### 2.5.2. EITC Analysis

The Spearman–Brown prophecy test was used to determine which item numbers could predict a Cronbach's coefficient >0.800, as this value is considered a preferable level of reliability [25, 26]. After determining the minimal item numbers, each item and the total score were transformed into dichotomous variables according to the three percentile levels. EITCs were calculated using Spearman's rho correlations, and the top-ranked items were rearranged in descending order of EITCs among the three percentile categories. As mentioned, one or two items with lower EITC levels were removed from the item pool with the top-ranked EITC, according to the total number of items calculated using the Spearman–Brown prophecy formula.

#### 2.5.3. Conduction of PCA

For the items within the eight factors, we examined which items resulted in a slight increase or minimal decrease in Cronbach's *α* of each factor [21]. Sequential removal of items was maintained until only two items remained for each factor. Since the “kidney-liver deficiency” factor of the 27-item YDS comprised only two items, the item removal procedure was not conducted for this factor. Through item reduction using the factor-maintaining method, 16 items, including two items in the eight factors, were determined for the short-form YDS. A PCA was conducted for the factor-based YDS comprising 16 items to examine whether there are any changes in the construct of the eight factors in the short-form YDS compared with that of the 27-item YDS. Only factors with eigenvalues greater than 1.0 were retained in PCA using the Kaiser criterion. Along with construct changes, we examined whether there were any changes in the overall Cronbach's *α* level for the 16-item YDS compared to that of the 27-item YDS.

#### 2.5.4. ROC Curve Analysis

After examining the reliability, construct validity, and dimensionality of the three short-form YDS using Rasch, EITC, and factor-based approaches, ROC curve analyses were conducted to compare their predictive accuracy for YD. In the three ROC curve analyses, the total scores of the short-form YDS served as test variables, and the presence or absence of YD, as determined by 12 clinicians in the previous study, served as the gold standard [1]. The predictive accuracy levels of the three short-form YDS were independently calculated using AUC. It is generally accepted that AUC values >0.9, 0.7–0.9, and 0.5–0.7 indicate high, moderate, and low accuracies, respectively [32]. An optimum cut-off point corresponded to the maximal Youden index (Youden index = sensitivity + specificity −1) [32].

#### 2.5.5. Correlation Analysis

A previous study reported that the 16-item KNQ consisted of four factors: neuropsychological, respiratory, neurogastrointestinal, and neuromuscular [20]. Correlations between the total and factor scores of the KNQ and factor-based YDS were examined using Pearson's rho coefficient. A correlation coefficient ≥0.8 was considered a “very strong correlation,” that of 0.6–0.7 was considered a “moderate correlation,” that of 0.3–0.5 was considered a “fair correlation,” and that of 0.1–0.2 was considered a “poor correlation” [33]. Correlation and factor analyses, reliability tests, and ROC curve analyses were performed using SPSS version 21 (SPSS Inc., Chicago, IL, USA), while Rasch analysis, including category probability, DIF, fitting error, unidimensionality, and person reliability index, was performed using Winsteps 4.8. Statistical significance was set at *P* < 0.05.

## 3. Results

### 3.1. Rasch Analysis

**Figure 1:**
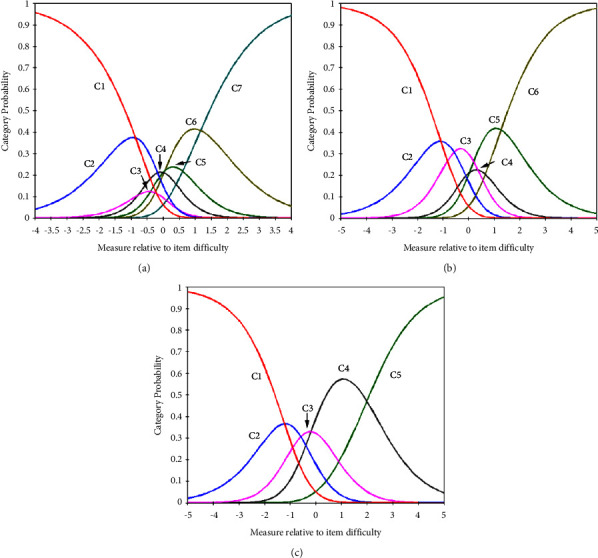
Probability curves of the 7-point (a), 6-point (b), and 5-point (c) responses. For the 7 points, C1 (category 1), disagree very strongly; C2 (category 2), disagree strongly; C3 (category 3), disagree; C4 (category 4), neither disagree nor agree; C5 (category 5), agree; C6 (category 6), agree strongly; and C7 (category 7), agree very strongly. In the 6 points, C3 (disagree) and C4 (neither disagree nor agree) of the 7 points were unified to C3 (disagree). In the 5 points, C4 (agree) and C5 (agree strongly) of the 6 points were unified to C4 (agree strongly).

The category characteristics of the 27 items are summarized in Table 2. The 7-point category responses were arranged in ascending order, and all outfit MnSq levels were below 2.0. The counts for the seven categories exceeded 10. However, the step calibration value for category 4 was lower than that for category 3, indicating that both categories were disordered.  Figure 1(a) shows the probability curve of Question 1 (Q1: night cough), according to a 7-point Likert scale, where the peak of category 3 was fused with that of category 4. This and the step calibration results indicated that categories 3 and 4 were disordered. Furthermore, the peak of category 5 sank under that of category 6, although the step calibration was slightly increased (0.04). This disordering between categories 3 and 4 and between 5 and 6 for the Q1 “night cough” were found equally for the probability curves of the other 26 question items. Categories 3 and 4 were first fused to correct for the categories' disordering because the degree of disordering of categories 3 and 4 was greater than that of categories 5 and 6. The fusion of categories 3 and 4 reduced seven categories to six (Table 2). The probability curve and step calibration analyses were repeated for the six categories. Consequently, the disordering of categories between categories 3 and 4 has been corrected. However, there was still the disordering of categories 4 and 5 among the six categories, which corresponded to the disordering of categories 5 and 6 among the seven categories (Figure 1(b)). This indicated that disorder existed among the six categories; categories 4 and 5 were unified, and the third-step calibration and probability curve analysis were conducted. Finally, 5-step categories showed an ascending order of step calibration throughout all categories, and all peaks of the probability curve were well separated (Figure 1(c)). This indicated that five response categories (disagree very strongly disagree strongly, disagree, agree strongly, and agree strongly) were suitable for respondents' answers to the YDS.

Table 3 presents the DIF results based on sex and age. The logit values for “afternoon fever (Q4),” “night fever (Q7),” “morning fatigue (Q15),” “susceptibility of heat and cold (Q16),” “night hot soles (Q22),” and “sweating during sleep (Q23)” in the older group were higher than those in the younger group, while the logit values for “persistent cough (Q2),” “residual urine (Q13),” “difficulty in containing the urine (Q14),” and “bone steaming (Q21)” in the younger group were higher than those in the older group. Regarding sex differences, the logit value only for “dark yellow urine (Q27)” in women was higher than in men. Therefore, 11 items showing DIF by age or sex were removed, and the remaining 16 were analyzed using Rasch analysis.

Table 4 lists the fit levels of the sixteen items without DIF. In the first analysis, “afternoon cough (Q3)” and “wake due to night urination (Q11)” showed fitting errors [18]. Therefore, the second analysis was conducted after removing the two items from the item pool. As a result, the remaining 14 items were free of fitting error, ranging in infit and outfit values from 0.70 to 1.39, and additional fitting analysis was not considered [18]. The raw or overall scores denoting the frequency of responses ranged from 341 points “night cough (Q1)” to 650 points “fatigue (Q17)”. In the reliability test, the person separation index was 2.19, and the person reliability was 0.83, indicating that the 14 items by Rasch analysis showed a high level of reliability [31].  Table 5 lists the dimensionality results of the 14 items. Unexplained variance in the first contrast was 1.994 (<2.0), implying the 14 items by Rasch analysis as unidimensional [22]. According to the category response, DIF, fitting error, and dimensionality analyses by Rasch analysis, the 14-item YDS rated on five categories was finally determined.

### 3.2. EITC Analysis

Table 6 lists the EITC results by three percentage points (25%, 50%, and 75%). In the Spearman–Brown prophecy analysis, 14 items were suggested as minimal numbers for guaranteeing Cronbach's *α* of 0.800. Therefore, in each percentile category, five items with top-ranked EITC values were extracted from the three percentile categories, respectively. Among the 15 items, “hair loss (Q25)” with the lowest EITC value (*r* = 0.368) was removed because the purpose of item shortening by EITC was to reduce items as many as possible while retaining Cronbach's *α* of 0.800. Finally, 14 items were determined as short-form YDS by ETIC. Cronbach's *α* for the 14-item YDS by EITC was 0.855.

### 3.3. Factor-Based Analysis

As mentioned earlier, items that contributed to a slight increase or minimal decrease in Cronbach's *α* values within a factor were removed item by item until two items remained within each factor. PCA was then conducted to examine the changes in the construct and Cronbach's *α* of the factors.  Table 7 lists the factor loadings of the 16 items and the Cronbach's *α* values of the factors. The eight factors in the 27-item YDS were reduced to five in the 16-item YDS. “Cough” and “fever” factors of the 27-item YDS were still preserved in the 16-item YDS, while “pain-weaknes” and “fatigue” factors of the 27-item YDS were unified into one in the 16-item YDS. Similarly, the “urine factor” and “skin-hair factor” were unified into one. The Cronbach's *α* values of the eight factors, which consisted of two items, ranged from 0.282 to 0.818 (Supplementary Table S1). However, the Cronbach's alpha for the five factors in the short-form YDS increased from 0.492 to 0.818. The total percentage of variance in the 16-item YDS by factor-based reduction was 61.61%, and the overall Cronbach's *α* of the 16 items was 0.828.

### 3.4. ROC Curve Analysis

Table 8 lists the ROC curve analyses of three short-form YDS versions by Rasch, EITC, and factor analyses. Supplementary Figure S1 shows maximal Youden points on the ROC curves of the three short-form YDS versions. The previous study has reported that the sensitivity, specificity, AUC, and cut-off points of the 27-item YDS were 78.7%, 84.8%, 0.885, and 10 points, respectively [1]. The AUC is a reflection how well the test distinguishes between YD and non-YD groups [32]. The AUC serves as a single measure summarizing the discriminative ability of a test across the full range of cut-offs, independently with the prevalence of disease or pathological pattern [34]. In this study, the AUC levels of 14-item Rasch, 14-item EITC, and 16-item factor-based YDS were 0.812, 0.811, and 0.818, indicating that three short-form YDS had moderate accuracy for determining YD.

**Figure 2:**
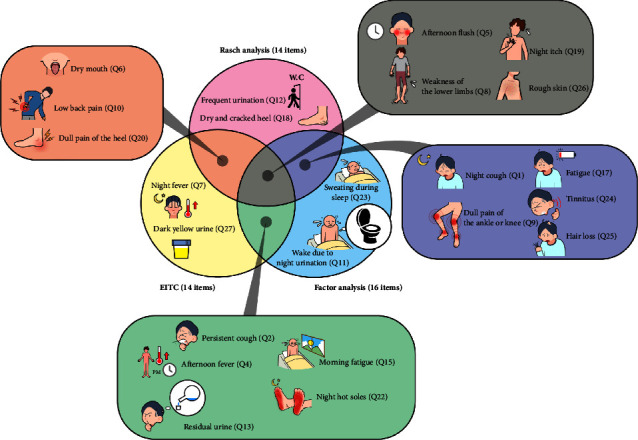
Items overlapped or separated by the three item-reduction methods. EITC, equidiscriminatory item-total correlation.

In examining sensitivity and specificity levels using the maximal Youden index, Rasch and factor-based models revealed similar sensitivity and specificity levels ranging from 0.737 to 0.789. However, for the EITC model, the sensitivity level (0.632) at the maximal Youden index (0.507) was lower than the specificity level (0.875), while the Youden index with similar sensitivity and specificity levels (0.719 and 0.723, respectively) was 0.443, being lower than the maximal Youden value of 0.507.  Figure 2 shows which items were overlapped or separated in the three short-form YDS versions. For example, “frequent urination (Q12)” and “dry and cracked heel (Q18)” were included only in the Rasch model, while “dry mouth (Q5),” “weakness of the lower limbs (Q8),” “night itch (Q19),” and “rough skin (Q26)” were overlapped with the three short-form YDS.

### 3.5. Correlation Analysis

In the examination of the incidence of YD among 237 college students, 51 students showed a total score of 27-item YDS over 10 points, and the incidence of YD was 21.5%.  Table 9 lists Pearson's correlations between the total and factor scores of the KNQ and the three short-form YDS versions. The total KNQ scores were positively correlated with the three short-form YDS by Rasch (*r* = 0.564), EITC (*r* = 0.498), and factor analysis (*r* = 0.517). The four-factor scores of the KNQ also showed fairly positive correlations with the total scores of the three short-form YDS versions (*r*; 0.352–0.489). About five-factor scores of the factor-based version, “fever,” “cough,” “sweating-feet,” and “urine-hair” had poor or fairly positive correlations with the total and the factor scores of the KNQ (*r*; 0.128–0.499).

## 4. Discussion

In this study, we developed three short-form YDS versions using the Rasch-, EITC-, and factor-based approaches. The main finding of this study was that the reliability and predictive accuracy of the three short-form YDS versions were comparable to those of the original 27-item YDS. This indicates that the 14-item Rasch and EITC YDS and the 16-item factor-based YDS can be utilized to estimate the severity of YD or determine the presence or absence of YD in clinical cases. However, our results also suggest that caution should be exercised when prioritizing the short-form YDS according to the characteristics of each approach, clinical situation, and study purpose.

Regarding the brevity of the three short-form YDS versions, the item reduction ratio of the Rasch and EITC approaches (13/27, both) was higher than that of the factor-based approach (11/27). Therefore, the short-form YDS by the Rasch and EITC approaches may be prioritized because the two questionnaires may shorten the completion time compared to the short-form YDS by a factor-based approach. In examining the reliability of the three short-form YDS versions, reliability levels estimated by Cronbach's *α* were preferable or higher. Interestingly, the final Cronbach's *α* of the EITC YDS was 0.855, which was higher than the value initially predicted by the Spearman–Brown prophecy formula (0.800). Although it was possible to reduce some items with lower EITC until Cronbach's *α* reached 0.800, we did not conduct additional item reduction by EITC because it might lower the predictive accuracy of the EITC YDS. Therefore, according to the Spearman–Brown prophecy formula, we determined the item number of the EITC YDS to be 14. Factor-based approaches are known to reduce the number of items while maintaining factor constructs [28]. By reducing items using the factor approach, an undesirable decrease in reliability within each factor was minimized because the items contributing to a slight increase or decrease in intrafactor reliability were primarily removed from the factor. The final Cronbach's *α* for the factor-based YDS was 0.827, indicating a preferable level of reliability [25]. In the Rasch approach, a higher person separation index denotes higher sensitivity in distinguishing between high and low respondents [14, 31]. This study's person separation index was 2.19, indicating a high-reliability level [31]. In summary, the three approaches to item reduction may not have significantly decreased the reliability of the original YDS.

In examining the predictive accuracy of the three short-form YDS versions, the AUC values of Rasch, EITC, and factor-based approaches were 0.812, 0.811, and 0.818, respectively. These values were considered as having “moderate accuracy” [32], and were similar to the AUC of 0.875 for the original YDS. Therefore, predictive accuracy equivalent to the original YDS may be expected when utilizing the 14-item Rasch and EITC YDS versions and the 16-item factor-based YDS version. However, it should be noted that among the three short-form YDS versions, the EITC YDS showed lower sensitivity (0.632) than specificity (0.875) at the maximal Youden points. One possibility is that the total scores of the 14 items in the EITC had a nonparametric distribution, which may have formed the jagged contour of the AUC. On the jagged contour, the increases or decreases in sensitivity and specificity tended to become irregular as the Youden index increases [35]. This means that for short-form YDS determined by the EITC, lower sensitivity or higher false-negative predictivity may have been barriers to the determination of YD using ROC curve analysis. Therefore, considering reliability, predictive accuracy, sensitivity, and specificity simultaneously, this study suggests using the short-form YDS version using Rasch and factor-based approaches rather than the EITC YDS.

Although both Rasch and factor-based versions showed satisfactory reliability and predictive accuracy, it should be emphasized that the short-form YDS by Rasch approach had a few advantages over the YDS by factor-based approach. Rasch analysis clarified the response category of the 27-item YDS by modifying the 7-point response scale of the original version of the YDS to five points. The response category of the five points of the short-form YDS was lower than that of the short-form Phlegm Pattern Questionnaire, where 6-point categories were finally determined using Rasch analysis [36]. After modifying the response category, 11 items with DIF regarding sex and age distribution and two items with infit or outfit errors were removed from the twenty-seven items of the YDS, and finally, fourteen items were determined. Among the fit indices, the outfit index was more sensitive to unexpected responses in items far from the person measure, whereas the infit index was more sensitive to unexpected responses in items close to the person measure [37]. Therefore, the short-form YDS from Rasch analysis may be broadly used in clinical cases, such as health checkups and epidemiological surveys, to minimize bias due to sex, aging, or unexpected responses.

In addition to the advantages of the Rasch approach, the advantages of the factor-based approach must also be described. This study showed “weak” positive correlations between the “cough” factor scores of the 16-item YDS and the “neuropsychological,” “respiratory,” and “neurogastrointestinal” factors of the KNQ. This suggests that the etiology of cough in YD may not be closely related to the etiology of dysfunctional breathing. Rather, the “fatigue” factor scores of YDS had “strong” positive correlations with the scores of “neuropsychological,” and “neurogastrointestinal” factors. This result may not guarantee the causality of YD-related fatigue with neuropsychological or neuro-gastrointestinal symptoms [13, 20]. Correlations between the factor scores of the factor-based YDS and the KNQ suggest that fatigue due to YD needs to be monitored and treated more intensively than other etiological or symptomatic factors of YD in patients with dysfunctional breathing. Therefore, the factor-based YDS may be used exclusively to examine YD's etiological, regional, and symptomatic characteristics in diverse diseases and syndromes.

This study had some limitations. Item reduction by DIF in Rasch analysis is affected by sample characteristics, including environmental or racial differences. Therefore, another item reduction of the YDS by Rasch analysis is needed in other samples to examine the similarity or dissimilarity of the 14-item YDS by Rasch analysis in this study. It should also be mentioned that the dataset used for item reduction in the original YDS was collected from outpatients who visited Korean medical clinics, whereas the dataset used to examine the correlation between YD and dysfunctional breathing was collected from a healthy young population. Therefore, it is necessary to examine the correlation between YD and dysfunctional breathing in the patient group. In the first dataset, there were more women (130 outpatients) than men (39 outpatients), which may have affected the results of the Rasch analysis. Further studies are needed to overcome these limitations regarding sample characteristics, healthy populations, and differences in the number of sexes.

## 5. Conclusions

This study aimed to develop three short-form YDS versions using Rasch, EITC, and factor-based approaches. Two datasets from previous studies (169 outpatients and 237 healthy college students) were analyzed. As a result, two types of the 14-item YDS were determined by Rasch and EITC analyses. A factor-based analysis suggested a 16-item YDS consisting of eight factors. The Rasch analysis suggested a 5-point response category to correct for the disordering of responses. The three-item reduction method showed moderate predictive accuracy in the ROC curve analysis. However, the specificity of the EITC method was lower than that of other item reduction methods. Factor scores of the short-form YDS were either weakly or strongly correlated with those of the KNQ. In conclusion, the 14-item Rasch YDS may be utilized to estimate YD's clinical severity or screen out YD for health checkups, primary care, or epidemiological surveys. In contrast, the 16-item Rasch YDS may be utilized to examine the relationship between the etiological factors of YD and other diseases.

## Figures and Tables

**Table 1:** The 27-item Yin Deficiency Scale.

Concept	Number	Item	Condition	Contribution score for yin deficiency (point)
Cough	Q1	I have a cough in the night	Night cough	5.96
	Q2	My cough lasts for a long time	Persistent cough	5.61
	Q3	I have a cough in the afternoon	Afternoon cough	4.88

Fever	Q4	I have a fever in the afternoon	Afternoon fever	5.22
	Q5	I have a flush in the afternoon	Afternoon flush	4.96
	Q6	I have a dry mouth	Dry mouth	4.88
	Q7	I have a fever in the night	Night fever	5.33

Pain-weakness	Q8	I feel heavy or weak in my lower limbs	Weakness of the lower limbs	4.59
	Q9	I feel dull pain in my ankle or knee	Dull pain of the ankle or knee	4.61
	Q10	I feel low back pain	Low back pain	5.40

Urine	Q11	I wake to urinate in the night	Wake due to night urination	5.25
	Q12	I urinate frequently	Frequent urination	5.07
	Q13	I feel a residual urine	Residual urine	4.44
	Q14	I cannot contain my urine	Difficulty in containing the urine	4.84

Fatigue	Q15	I feel tired in the morning	Morning fatigue	5.33
	Q16	I am susceptible of heat and cold	Susceptibility of heat and cold	6.07
	Q17	I feel tired or languid	Fatigue	4.96

Feet-bone steaming	Q18	My heel is dry and cracked	Dry and cracked heel	5.37
	Q19	I have an itch in the night	Night itch	5.37
	Q20	I feel dull pain in my heel	Dull pain of the heel	5.11
	Q21	I feel hot deep in the body, e.g., in the bone	Bone steaming	5.38
	Q22	My soles are hot in the night	Night hot soles	6.00

Kidney-liver deficiency	Q23	I sweat during sleep	Sweating during sleep	5.33
	Q24	My ear rings	Tinnitus	5.29

Skin-hair	Q25	My hair falls out	Hair loss	4.77
	Q26	I have a rough skin	Rough skin	5.03
	Q27	My urine color is dark yellow	Dark yellow urine	4.74

Contribution scores were previously reported through two iterations of the Delphi method [27].

**Table 2:** Category characteristics of the 7-, 6-, and 5-point Likert scales.

Scale	Category (response)	Observed count (%)	Average measure	Expected measure	Infit MnSq	Outfit MnSq	Step calibration	Category measure
7-point Likert	1 (disagree very strongly)	992 (22)	−0.89	−0.83	0.93	1.00	None	(−2.18)
	2 (disagree strongly)	1141 (25)	−0.59	−0.63	1.00	0.97	−0.87	−0.94
	3 (disagree)	428 (9)	−0.32	−0.44	1.05	0.89	0.45	−0.43
	4 (neither disagree nor agree)	667 (15)	−0.25	−0.26	0.97	0.97	−0.79^*∗*^	−0.08
	5 (agree)	567 (12)	−0.12	−0.08	1.10	1.16	0.00	0.31
	6 (agree strongly)	554 (12)	0.09	0.12	1.08	1.17	0.04	0.98
	7 (agree very strongly)	214 (5)	0.31	0.31	1.07	1.09	1.17	(2.48)

6-point Likert	1 (disagree very strongly)	992 (22)	−1.19	−1.12	0.96	0.99	None	(−2.50)
	2 (disagree strongly)	1141 (25)	−0.74	−0.77	0.92	0.87	−1.08	−1.10
	3 (disagree)	1095 (24)	−0.38	−0.45	0.95	0.89	−0.57	−0.31
	4 (agree)	567 (12)	−0.17	−0.15	1.06	1.10	0.36	0.30
	5 (agree strongly)	554 (12)	0.12	0.16	1.10	1.18	0.03^*∗*^	1.07
	6 (agree very strongly)	214 (5)	0.38	0.46	1.16	1.21	1.26	(2.56)

5-point Likert	1 (disagree very strongly)	992 (22)	−1.37	−1.31	0.96	1.01	None	(−2.61)
	2 (disagree strongly)	1141 (25)	−0.81	−0.85	0.92	0.87	−1.22	−1.19
	3 (disagree)	1095 (24)	−0.34	−0.40	0.89	0.88	−0.58	−0.21
	4 (agree strongly)	1121 (25)	0.04	0.08	1.10	1.16	−0.19	1.08
	5 (agree very strongly)	214 (5)	0.52	0.59	1.13	1.10	1.99	(3.17)

^*∗*^Disordering response category, where step calibration value became lower than that of the previous category. MnSq, mean square.

**Table 3:** Differential item functioning results by sex and age.

Item (no.)	Sex				Age			
	Male (logit)	Female (logit)	Chi-square value	*P* value	Younger (logit)	Older (logit)	Chi-square value	*P* value
Night cough (Q1)	0.67	0.76	0.142	0.706	0.85	0.63	1.814	0.178
Persistent cough (Q2)	0.70	0.48	3.355	0.067	0.72	0.35	5.907	**0.015**
Afternoon cough (Q3)	0.87	0.92	0.272	0.602	1.05	0.81	0.426	0.514
Afternoon fever (Q4)	0.33	0.56	0.711	0.399	0.36	0.65	4.260	**0.039**
Afternoon flush (Q5)	0.43	0.43	0.006	0.940	0.33	0.54	2.301	0.129
Dry mouth (Q6)	−0.24	−0.24	0.399	0.528	−0.10	−0.37	1.195	0.274
Night fever (Q7)	0.24	0.42	0.945	0.331	0.19	0.55	6.495	**0.011**
Weakness of the lower limbs (Q8)	−0.11	−0.15	0.949	0.330	−0.05	−0.25	0.134	0.715
Dull pain of the ankle or knee (Q9)	0.06	−0.39	3.044	0.081	−0.24	−0.33	0.248	0.618
Low back pain (Q10)	−0.78	−0.78	0.015	0.902	−0.66	−0.90	0.063	0.802
Wake due to night urination (Q11)	−0.24	−0.24	0.018	0.893	−0.15	−0.32	2.847	0.091
Frequent urination (Q12)	−0.70	−0.48	1.464	0.226	−0.53	−0.53	0.036	0.850
Residual urine (Q13)	−0.05	0.34	1.377	0.241	0.50	0.02	4.509	**0.034**
Difficulty in containing the urine (Q14)	0.67	0.60	0.012	0.914	0.87	0.38	7.705	**0.006**
Morning fatigue (Q15)	−0.96	−1.19	0.292	0.589	−1.48	−0.82	6.700	**0.010**
Susceptibility of heat and cold (Q16)	−0.61	−0.97	1.488	0.223	−1.15	−0.64	5.001	**0.025**
Fatigue (Q17)	−1.27	−1.54	1.199	0.274	−1.35	−1.60	1.424	0.233
Dry and cracked heel (Q18)	0.12	−0.02	0.281	0.596	−0.07	0.09	0.007	0.932
Night itch (Q19)	0.24	0.30	0.017	0.896	0.27	0.33	0.882	0.348
Dull pain of the heel (Q20)	0.30	0.33	0.435	0.510	0.33	0.33	0.118	0.731
Bone steaming (Q21)	0.77	0.82	0.886	0.347	0.99	0.68	6.234	**0.013**
Night hot soles (Q22)	0.48	0.18	0.241	0.624	0.09	0.41	7.401	**0.007**
Sweating during sleep (Q23)	−0.02	0.20	0.923	0.337	0.06	0.24	4.871	**0.027**
Tinnitus (Q24)	0.18	0.04	0.036	0.850	0.18	−0.03	0.467	0.494
Hair loss (Q25)	0.03	0.03	2.309	0.129	−0.16	0.21	0.010	0.922
Rough skin (Q26)	−0.22	−0.22	0.160	0.689	−0.36	−0.09	3.697	0.055
Dark yellow urine (Q27)	−0.76	−0.25	5.288	**0.022**	−0.40	−0.32	0.307	0.580

The *P* values in bold indicate significant differences in logit values between sexes or higher and lower age groups.

**Table 4:** Item difficulty and fitting levels of the short-form Yin Deficiency Scale using Rasch analysis.

	Item (no.)	Raw score	Model measure	Infit MnSq	Outfit MnSq
First analysis	Night cough (Q1)	341	0.79	0.94	0.99
	Afternoon cough (Q3)	318	0.97	0.72	**0.67**
	Afternoon flush (Q5)	383	0.49	0.83	0.81
	Dry mouth (Q6)	486	−0.16	1.04	1.02
	Weakness of the lower limbs (Q8)	473	−0.08	0.78	0.78
	Dull pain of the ankle or knee (Q9)	494	−0.22	1.06	1.06
	Low back pain (Q10)	566	−0.70	0.97	0.97
	Wake due to night urination (Q11)	486	−0.16	**1.40**	**1.46**
	Frequent urination (Q12)	530	−0.45	1.13	1.18
	Fatigue (Q17)	650	−1.39	1.16	1.18
	Dry and cracked heel (Q18)	448	0.08	1.09	1.12
	Night itch (Q19)	403	0.36	0.94	0.96
	Dull pain of the heel (Q20)	399	0.39	0.85	0.85
	Tinnitus (Q24)	438	0.14	1.32	1.32
	Hair loss (Q25)	445	0.09	1.02	1.07
	Rough skin (Q26)	484	−0.15	0.83	0.87

The second analysis	Night cough (Q1)	341	0.86	1.02	1.06
	Afternoon flush (Q5)	383	0.56	0.86	0.83
	Dry mouth (Q6)	486	−0.11	1.06	1.05
	Weakness of the lower limbs (Q8)	473	−0.03	0.75	0.75
	Dull pain of the ankle or knee (Q9)	494	−0.16	1.02	1.04
	Low back pain (Q10)	566	−0.65	0.97	0.95
	Frequent urination (Q12)	530	−0.40	1.19	1.24
	Fatigue (Q17)	650	−1.35	1.15	1.16
	Dry and cracked heel (Q18)	448	0.13	1.10	1.13
	Night itch (Q19)	403	0.43	0.96	0.99
	Dull pain of the heel (Q20)	399	0.45	0.83	0.84
	Tinnitus (Q24)	438	0.20	1.31	1.29
	Hair loss (Q25)	445	0.15	1.03	1.12
	Rough skin (Q26)	484	−0.10	0.81	0.85

MnSq, mean square. Bold letters indicate infit or outfit MnSq under 0.70 or over 1.40.

**Table 5:** Dimensionality results of the 14-item Yin Deficiency Scale by Rasch analysis.

Standardized residual variance	Eigenvalue (%)
Total raw variance in observations	23.201 (100)
Raw variance explained by measures	9.208 (39.7)
Raw variance explained by persons	2.851 (12.3)
Raw variance explained by items	6.357 (27.4)
Raw unexplained variance (total)	14.000 (60.3)
Unexplained variance in 1^st^ contrast	**1.994** (8.6)
Unexplained variance in 2^nd^ contrast	1.770 (7.6)
Unexplained variance in 3^rd^ contrast	1.485 (6.4)
Unexplained variance in 4^th^ contrast	1.335 (5.8)
Unexplained variance in 5^th^ contrast	1.261 (5.4)

The value in bold indicates an acceptable eigenvalue of the variance for unidimensionality (<2.0).

**Table 6:** Equidiscriminatory item-total correlation results by three percentile points.

25% cut-off points of the total scores (72 points)	Spearman correlation	50% cut-off points of the total scores (88 points)	Spearman correlation	75% cut-off points of the total scores (104 points)	Spearman correlation
Dry mouth^a^	0.500	Weakness of the lower limbs^a^	0.555	Night fever	0.531
Night fever^a^	0.477	Low back pain	0.548	Afternoon fever	0.528
Low back pain^a^	0.477	Afternoon flush^a^	0.529	Night itch^a^	0.505
Night hot soles^a^	0.473	Night fever	0.523	Afternoon flush	0.503
Afternoon fever^a^	0.467	Dull pain in the heel^a^	0.523	Rough skin^a^	0.491
Weakness of the lower limbs	0.450	Afternoon fever	0.521	Night hot soles	0.471
Sweating during sleep	0.446	Persistent cough^a^	0.509	Weakness of the lower limbs	0.433
Residual urine	0.440	Residual urine^a^	0.504	Residual urine	0.420
Bone steaming	0.437	Night hot soles	0.497	Dark yellow urine^a^	0.409
Dull pain in the ankle or knee	0.436	Night itch	0.491	Dry mouth	0.380
Afternoon flush	0.435	Dull pain in the ankle or knee	0.457	Morning fatigue^a^	0.371
Fatigue	0.424	Bone steaming	0.451	Hair loss^a^	0.368
Dull pain in the heel	0.422	Morning fatigue	0.434	Dull pain in the heel	0.367
Afternoon cough	0.410	Sweating during sleep	0.432	Tinnitus	0.348
Night itch	0.384	Afternoon cough	0.430	Persistent cough	0.339
Dry and cracked heel	0.361	Night cough	0.428	Waking up due to night urination	0.325
Night cough	0.355	Fatigue	0.420	Afternoon cough	0.324
Dark yellow urine	0.338	Rough skin	0.420	Low back pain	0.323
Rough skin	0.331	Dark yellow urine	0.403	Night cough	0.322
Morning fatigue	0.328	Susceptibility to heat and cold	0.398	Dull pain in the ankle or knee	0.320
Persistent cough	0.326	Dry mouth	0.386	Sweating during sleep	0.318
Tinnitus	0.324	Hair loss	0.333	Susceptibility to heat and cold	0.313
Difficulty containing urine	0.303	Waking up due to night urination	0.322	Difficulty containing urine	0.310
Susceptibility to heat and cold	0.301	Difficulty containing urine	0.315	Frequent urination	0.307
Waking up due to night urination	0.293	Tinnitus	0.307	Fatigue	0.299
Hair loss	0.282	Frequent urination	0.294	Dry and cracked heel	0.296
Frequent urination	0.276	Dry and cracked heel	0.294	Bone steaming	0.276

^a^15 items by summation of five top-ranked items among three percentile categories. All Spearman correlations had *P* < 0.01. Among the 15 items, “hair loss” had lowest EITC (0.368) and was finally removed.

**Table 7:** Factor loadings of sixteen items and Cronbach's *α* values of the factors.

Item	Cronbach's *α* of each factor	Factor				
		1	2	3	4	5
Afternoon flush	0.737	**0.736**	0.368	0.048	0.027	0.128
Rough skin		**0.734**	−0.051	0.264	0.100	0.054
Afternoon fever		**0.688**	0.253	0.107	0.240	0.107

Persistent cough	0.818	0.112	**0.838**	0.135	0.176	0.015
Night cough		0.135	**0.811**	0.025	0.208	0.072

Morning fatigue	0.724	0.061	0.064	**0.783**	0.138	0.106
Fatigue		0.108	0.153	**0.769**	−0.174	0.295
Dull pain of the ankle or knee		0.255	−0.048	**0.614**	0.408	−0.202
Weakness of the lower limbs		0.397	0.106	**0.501**	0.435	−0.064

Sweating during sleep	0.615	−0.067	0.154	0.127	**0.675**	0.164
Night hot soles		0.376	0.151	0.003	**0.632**	0.153
Night itch		0.362	0.238	0.048	**0.581**	−0.051

Wake due to night urination	0.492	0.057	0.303	0.061	−0.006	**0.588**
Hair loss		0.444	−0.207	0.001	0.136	**0.576**
Tinnitus		−0.109	−0.122	0.280	0.383	**0.545**
Residual urine		0.311	0.468	0.042	0.040	**0.473**

Variance explained (%)		15.05	12.97	12.73	11.92	8.94

Bold letters indicate maximal factor loadings among the five factors. Factor 1, fever factor; factor 2, cough factor; factor 3, fatigue-pain-weakness factor; factor 4, sweating-feet factor; factor 5, urine-hair factor. Final overall Cronbach's *α* of the 16 items was 0.828.

**Table 8:** ROC curve analyses of the three short-form YDS by Rasch, EITC, and factor-based approaches.

Rasch analysis (item numbers = 14)				EITC (item numbers = 14)				Factor analysis (item numbers = 16)			
Points	Sensitivity	Specificity	Youden index	Points	Sensitivity	Specificity	Youden index	Points	Sensitivity	Specificity	Youden index
29.5	0.965	0.205	0.170	42.5	0.825	0.589	0.414	44.5	0.895	0.438	0.332
30.5	0.965	0.232	0.197	43.5	0.807	0.607	0.414	45.5	0.877	0.473	0.350
31.5	0.947	0.277	0.224	44.5	0.789	0.634	0.423	46.5	0.860	0.509	0.369
32.5	0.947	0.321	0.269	45.5	0.772	0.652	0.424	47.5	0.860	0.545	0.404
33.5	0.930	0.384	0.314	46.5	0.737	0.679	0.415	48.5	0.860	0.598	0.458
34.5	0.930	0.464	0.394	47.5	0.719	0.723	0.443	49.5	0.860	0.616	0.476
35.5	0.930	0.482	0.412	48.5	0.684	0.768	0.452	50.5	0.789	0.634	0.423
36.5	0.895	0.509	0.404	49.5	0.667	0.795	0.461	51.5	0.789	0.661	0.450
37.5	0.860	0.563	0.422	51.0	0.667	0.813	0.479	52.5	0.789	0.679	0.468
38.5	0.842	0.634	0.476	52.5	0.632	0.848	0.480	53.5	0.772	0.688	0.459
39.5	0.807	0.705	0.512	**53.5**	0.632	0.875	**0.507**	54.5	0.754	0.750	0.504
**40.5**	0.789	0.741	**0.531**	54.5	0.596	0.893	0.489	55.5	0.754	0.768	0.522
41.5	0.702	0.759	0.461	55.5	0.561	0.902	0.463	**56.5**	0.737	0.786	**0.523**
42.5	0.684	0.795	0.479	56.5	0.509	0.902	0.411	57.5	0.684	0.813	0.497
43.5	0.596	0.848	0.445	57.5	0.491	0.920	0.411	58.5	0.649	0.830	0.479
44.5	0.509	0.866	0.375	58.5	0.439	0.929	0.367	59.5	0.632	0.848	0.480
45.5	0.439	0.902	0.340	59.5	0.421	0.955	0.376	60.5	0.614	0.848	0.462
46.5	0.368	0.946	0.315	60.5	0.351	0.955	0.306	61.5	0.579	0.857	0.436

ROC, receiver operating characteristic; YDS, Yin Deficiency Scale; EITC, equidiscriminatory item-total correlation. The values in bold indicate maximum Youden indexes (sensitivity + specificity− 1) obtained using ROC curve analyses and cut-off points corresponding to the Youden indexes.

**Table 9:** Correlations between the total and factor scores of the KNQ and the three short-form YDS versions.

KNQ		Total scores of the 14-item YDS by Rasch analysis	Total scores of the 14-item YDS by EITC	16-item YDS by factor analysis					
				Total scores	Factors				
					Fever	Cough	Fatigue-pain-weakness	Sweating-feet	Urine-hair
Total scores		0.564^*∗∗*^	0.498^*∗∗*^	0.517^*∗∗*^	0.363^*∗∗*^	0.211^*∗∗*^	0.499^*∗∗*^	0.379^*∗∗*^	0.380^*∗∗*^

Factors	Neuropsychological	0.489^*∗∗*^	0.448^*∗∗*^	0.457^*∗∗*^	0.331^*∗∗*^	0.187^*∗∗*^	0.447^*∗∗*^	0.316^*∗∗*^	0.335^*∗∗*^
	Respiratory	0.426^*∗∗*^	0.389^*∗∗*^	0.394^*∗∗*^	0.295^*∗∗*^	0.158^*∗*^	0.352^*∗∗*^	0.320^*∗∗*^	0.282^*∗∗*^
	Neuro-gastrointestinal	0.447^*∗∗*^	0.352^*∗∗*^	0.378^*∗∗*^	0.245^*∗∗*^	0.128^*∗*^	0.408^*∗∗*^	0.239^*∗∗*^	0.293^*∗∗*^
	Neuromuscular	0.405^*∗∗*^	0.385^*∗∗*^	0.407^*∗∗*^	0.267^*∗∗*^	0.219^*∗∗*^	0.349^*∗∗*^	0.361^*∗∗*^	0.284^*∗∗*^

KNQ, the Korean version of the Nijmegen Questionnaire; YDS, Yin Deficiency Scale; EITC, equidiscriminatory item-total correlation. ^*∗*^*P* < 0.05. ^*∗∗*^*P* < 0.01.

## Data Availability

The data used to support the findings of this study have not been made available because they include personal information.

## Supplementary Materials

Supplementary Materials Supplementary Table S1: eight factors of the 27-item Yin Deficiency Scale. Supplementary Figure S1: ROC curves of the three short-form YDS versions and maximal Youden points. ROC, receiver operator characteristics; YDS, Yin Deficiency Scale; EITC, equidiscriminatory item-total correlation. A: ROC curve of the 14-item YDS using the Rasch approach; B: ROC curve of the 14-item YDS using the EITC; C: ROC curve of the 16-item YDS using factor analysis. In each ROC curve, the red dot corresponds to the point of maximal Youden index.
