# Supplementary material for: Development of the Short-Form Yin Deficiency Scale Using Three Item Reduction Approaches
Source: Evid Based Complement Alternat Med. 2024 Jan 19;2024:5533815. doi: 10.1155/2024/5533815 (PMC10817808; doi:10.1155/2024/5533815)
Supplement: Supplementary Materials — Supplementary Table S1: eight factors of the 27-item Yin Deficiency Scale. Supplementary Figure S1: ROC curves of the three short-form YDS versions and maximal Youden points. ROC, receiver operator characteristics; YDS, Yin Deficiency Scale; EITC, equidiscriminatory item-total correlation. A: ROC curve of the 14-item YDS using the Rasch approach; B: ROC curve of the 14-item YDS using the EITC; C: ROC curve of the 16-item YDS using factor analysis. In each ROC curve, the red dot corresponds to the point of maximal Youden index. [file 5533815.f1.zip › Supplemental_Table 1.docx]

Supplemental Table 1. Extraction of sixteen items from the eight factors of the 27-item Yin-Deficiency Scale

| Item | Cronbach’s α of all items (Cronbach’s α of two items) | Factor | | | | | | | |
| --- | --- | --- | --- | --- | --- | --- | --- | --- | --- |
|  |  | 1 | 2 | 3 | 4 | 5 | 6 | 7 | 8 |
| Night cough* | 0.846  (0.818) | **0.840** | 0.123 | 0.027 | 0.140 | 0.020 | 0.136 | 0.095 | 0.048 |
| Persistent cough* |  | **0.822** | 0.171 | 0.069 | 0.109 | 0.176 | 0.093 | 0.037 | -0.098 |
| Afternoon cough |  | **0.785** | 0.232 | 0.085 | 0.107 | -0.018 | 0.110 | 0.074 | 0.109 |
| Afternoon fever* | 0.807  (0.784) | 0.228 | **0.760** | 0.230 | 0.021 | 0.010 | 0.072 | 0.184 | 0.173 |
| Afternoon flush* |  | 0.289 | **0.738** | 0.052 | 0.084 | 0.091 | 0.165 | -0.035 | 0.249 |
| Dry mouth |  | 0.115 | **0.703** | 0.110 | 0.300 | 0.172 | 0.061 | 0.047 | -0.098 |
| Night fever |  | 0.123 | **0.604** | 0.129 | -0.008 | -0.048 | 0.225 | 0.540 | 0.132 |
| Weakness of the lower limbs* | 0.784  (0.772) | 0.205 | 0.167 | **0.812** | 0.033 | 0.116 | 0.108 | 0.055 | 0.175 |
| Dull pain of the ankle or knee* |  | 0.009 | 0.161 | **0.751** | -0.080 | 0.312 | 0.170 | 0.071 | -0.094 |
| Low back pain |  | -0.045 | 0.155 | **0.641** | 0.231 | 0.188 | 0.090 | 0.199 | 0.088 |
| Wake due to night urination* | 0.618  (0.528) | 0.008 | 0.171 | -0.050 | **0.676** | 0.118 | 0.191 | 0.088 | -0.079 |
| Frequent urination |  | 0.145 | 0.019 | 0.031 | **0.621** | 0.096 | -0.216 | 0.231 | 0.217 |
| Residual urine* |  | 0.308 | 0.261 | 0.084 | **0.569** | 0.072 | 0.141 | -0.035 | 0.242 |
| Difficulty containing urine |  | 0.150 | -0.061 | 0.266 | **0.553** | -0.002 | 0.192 | -0.169 | 0.084 |
| Morning fatigue* | 0.671  (0.695) | 0.078 | -0.007 | 0.265 | 0.039 | **0.724** | -0.015 | 0.250 | 0.118 |
| Susceptibility to heat and cold |  | 0.104 | 0.035 | 0.027 | 0.036 | **0.703** | 0.284 | 0.012 | 0.190 |
| Fatigue* |  | 0.023 | 0.205 | 0.222 | 0.245 | **0.682** | -0.113 | 0.043 | -0.050 |
| Dry and cracked heel | 0.702  (0.597) | 0.022 | 0.227 | -0.012 | 0.146 | 0.138 | **0.684** | 0.052 | -0.126 |
| Night itch* |  | 0.320 | 0.082 | 0.252 | -0.049 | 0.090 | **0.555** | 0.143 | 0.225 |
| Dull pain of the heel |  | 0.141 | 0.054 | 0.342 | 0.156 | 0.031 | **0.483** | 0.075 | 0.240 |
| Bone steaming |  | 0.188 | -0.018 | 0.417 | 0.240 | -0.210 | **0.483** | 0.027 | 0.138 |
| Night hot soles* |  | 0.153 | 0.364 | 0.152 | -0.085 | 0.030 | **0.469** | 0.450 | 0.169 |
| Sweating during sleep* | 0.282  (0.282) | 0.183 | 0.102 | 0.017 | -0.033 | 0.188 | 0.189 | **0.690** | 0.025 |
| Tinnitus* |  | -0.071 | -0.008 | 0.314 | 0.274 | 0.066 | -0.068 | **0.522** | 0.087 |
| Hair loss* | 0.533  (0.521) | -0.058 | 0.103 | 0.057 | 0.157 | 0.044 | 0.132 | 0.106 | **0.725** |
| Rough skin* |  | 0.033 | 0.404 | 0.227 | -0.069 | 0.321 | 0.145 | -0.151 | **0.559** |
| Dark yellow urine |  | 0.332 | 0.084 | 0.060 | 0.183 | 0.129 | -0.115 | 0.308 | **0.496** |

The items marked with asterisks are the two items that finally remained after removing the items with lower Cronbach’s α from each factor. Bold letters indicate the highest factor loadings of the items. As the 7th factor consisted of two items, the item reduction was not applied. Factor 1; cough factor, Factor 2; fever factor, Factor 3; pain-weakness factor, Factor 4; urine factor, Factor 5; fatigue factor, Factor 6; feet-bone steaming factor, Factor 7; kidney-liver deficiency factor, Factor 8; skin-hair factor.
