# Supplementary figures and images for: Development of the Short-Form Yin Deficiency Scale Using Three Item Reduction Approaches
Source: Evid Based Complement Alternat Med. 2024 Jan 19;2024:5533815. doi: 10.1155/2024/5533815 (PMC10817808; doi:10.1155/2024/5533815)

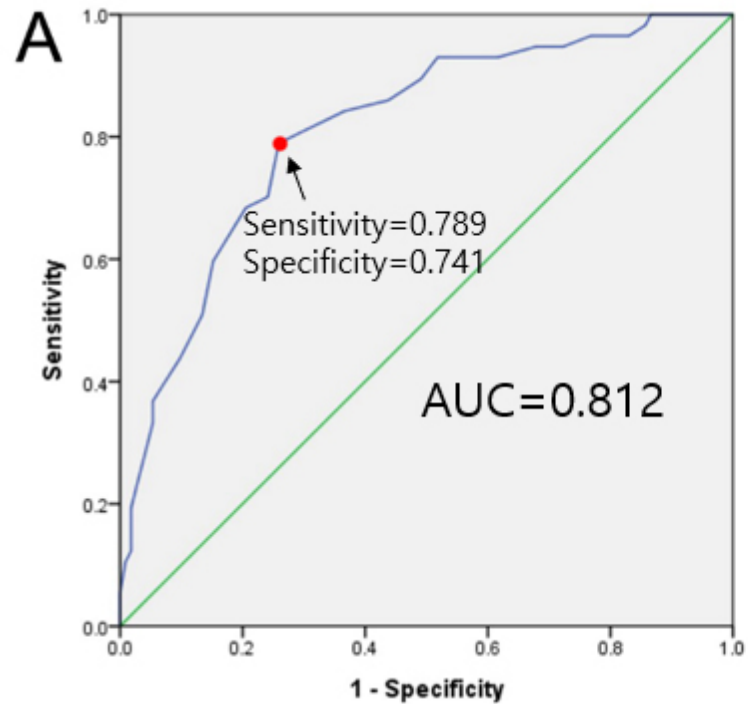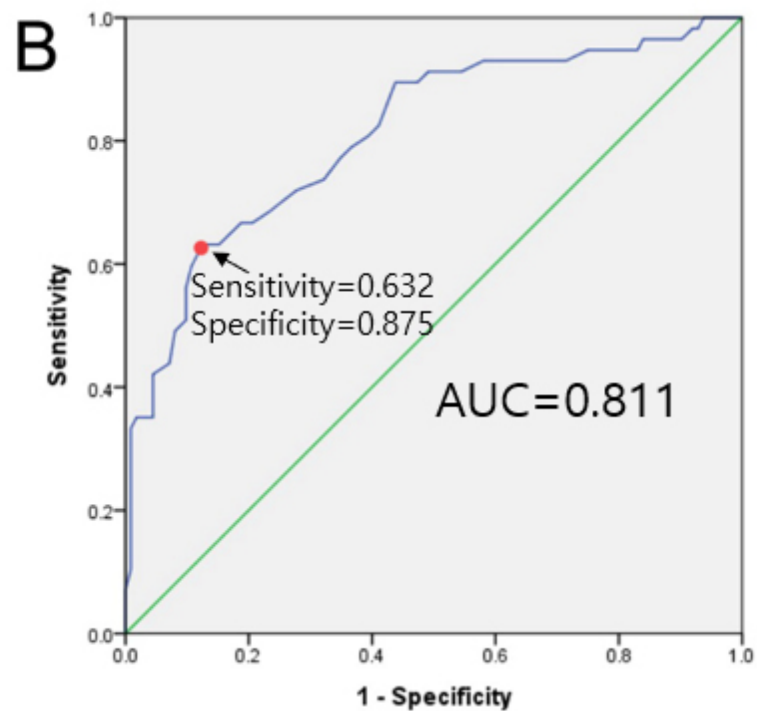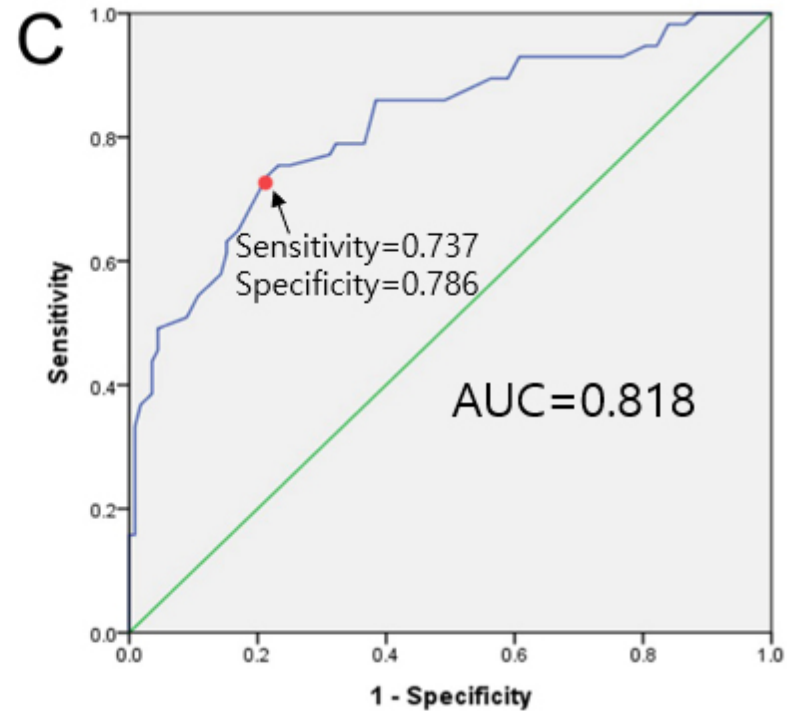

Supplement: Supplementary Materials — Supplementary Table S1: eight factors of the 27-item Yin Deficiency Scale. Supplementary Figure S1: ROC curves of the three short-form YDS versions and maximal Youden points. ROC, receiver operator characteristics; YDS, Yin Deficiency Scale; EITC, equidiscriminatory item-total correlation. A: ROC curve of the 14-item YDS using the Rasch approach; B: ROC curve of the 14-item YDS using the EITC; C: ROC curve of the 16-item YDS using factor analysis. In each ROC curve, the red dot corresponds to the point of maximal Youden index. [file 5533815.f1.zip › Supplemental Figure 1.pdf]
